# A Homeodomain Transcription Factor Gene, *PfMSX*, Activates Expression of *Pif* Gene in the Pearl Oyster *Pinctada fucata*

**DOI:** 10.1371/journal.pone.0103830

**Published:** 2014-08-06

**Authors:** Mi Zhao, Maoxian He, Xiande Huang, Qi Wang

**Affiliations:** 1 CAS Key Laboratory of Tropical Marine Bio-resources and Ecology, South China Sea Institute of Oceanology, Chinese Academy of Sciences, Guangzhou, China; 2 University of Chinese Academy of Sciences, Beijing, China; Ecole Normale Supérieure de Lyon, France

## Abstract

We reported pearl oyster *Pinctada fucata* cDNA and genomic characterization of a new homeobox-containing protein, PfMSX. The *PfMSX* gene encodes a transcription factor that was localized to the nucleus. Analyses of *PfMSX* mRNA in tissues and developmental stages showed high expressions in mantle or D-shaped larvae. In electrophoretic mobility shift assays (EMSAs) PfMSX binded to MSX consensus binding sites in the 5′ flanking region of the *Pif* promoter. In co-transfection experiment PfMSX transactivated reporter constructs containing *Pif* promoter sequences, and mutation of the MSX-binding sites attenuated transactivation. A knockdown experiment using *PfMSX* dsRNA showed decreased *Pif* mRNA and unregular crystallization of the nacreous layer using scanning electron microscopy. Our results suggested that *PfMSX* was a conserved homeodomain transcription factor gene, which can activate *Pif* gene expression through MSX binding site, and was then involved in the mineralization process in pearl oyster *Pinctada fucata*. Our data provided important clues about mechanisms regulating biomineralization in pearl oyster.

## Introduction

Pearl oyster, *Pinctada fucata,* widely distributed in tropic and subtropic marine coasts, is an important marine bivalve species that is cultured for pearl production and hot research topics in biomineralization, for its highly organized internal structure, chemical complexity, mechanical properties and optical effects of nacre [1]. *P. fucata* can generate the shell in vivo consisting of two different forms of calcium carbonate, i.e. aragonite in the inner nacreous layer and calcite in the outer prismatic layer. The organic matrix, secreted by the mantle epithelium, comprises 1–5% of the shell weight. At molecular level, the matrix proteins play a key role in the mineralization process, which is thought to direct the growth of calcium carbonate crystal and be responsible for the extraordinary properties of nacre and prism [2]–[6], but the molecular and cellular regulatory mechanisms underlying its biomineralization remain largely unknown.

The MSX gene family is one of the oldest animal-specific homeodomain transcription factors which are confined to the Metazoa [7]. MSX genes are found in animals ranging from sponge to mammal [8]–[11]. Expansion of the MSX family in amphibia and vertebrates via gene duplication has been accompanied by divergent expression patterns between MSX paralogs [12]. While most of invertebrates and primitive chordates examined to date have single MSX-like genes except anthozoas [10] and planarians [13].

Although *MSX* genes have been isolated from major metazoan phyla [14], the role of *MSX* genes in each animal taxa is very limited except in mammals. Molecular phylogenetic analysis of wide-ranging groups of metazoan animals indicated that functionally important residues in MSX proteins are strongly conserved in all metazoan MSX homeodomains which is known to physically interact with DNAs or proteins that are essential for the molecular function [15]. More studies have shown MSX in vertebrates is a homeodomain transcription factor implicated in a variety of developmental processes from tooth formation to limb regeneration and development [16]–[18]. For example, mutations in mice MSX1 result in craniofacial abnormalities that include cleft palate and absence of specific teeth [19], [20], while mice expressing a mutated MSX2 transgene exhibit the symptoms of craniosynostosis, a disease characterized by premature closure of the cranial sutures [16], [21], more severely, double mutants of MSX1and MSX2 exhibit a severe limb phenotype [22].

Homeodomain genes are among the most slowly evolving of all protein classes [23], and the amino acid sequences of DNA binding domains are usually highly conserved [24]. Likewise, most transcription factor sequence preferences are thought to be largely unchanged over vast evolutionary distances [25]. The high degree of evolutionary conservation of the homeodomain suggests that one of the regulatory circuits may be the same [26]. Pif, is a key biomineralization-related matrix protein for nacre formation in *Pinctada fucata* [27]. We amplified its 5′ flanking sequence and found two suspicious homeodomain binding site “ACTAATTGG” between –1008 and –1000 (transcriptional initiation site is defined as +1) and “GTAATTG” between −412 and −406 (termed as MBS (MSX binding site)-1 and MBS-2 respectively) which are identical to the consensus MSX binding site “G/CTAATTG” in mice or humans [28], [29]. We next cloned and identified a MSX-homologous gene in the pearl oyster *Pinctada fucata*, designated as *PfMSX*, which is highly conserved in its homeodomain comparing with other known mammalian MSX proteins. These findings led us to speculate that *PfMSX* could be a transcription factor of the *Pif* gene, which may bind to the consensus MSX-binding site of the *Pif* promoter, further governs the expression of *Pif* gene and control the biomineralization of the shell formation.

To address these hypotheses, we identified an ancestral conserved homeobox-containing gene *PfMSX* which recognized a conserved MSX-binding site and played an important role in the *Pif* expression in *Pinctada fucata*.

Adult pearl oysters (shell length 4.5–5.5 cm) were sampled from Daya Bay Station (China Marine Biology Research Station, South China Sea Institute of Oceanology, the Chinese Academy of Sciences) in Shenzhen, China. The field studies did not involve endangered or protected species. The specific location of our study: (longitude: 114.533624; latitude: 22.556406 north).

## Materials and Methods

### Cloning of *PfMSX* full-length cDNA and genomic structure

Gigabase-scale transcriptomes sequencing, assembly and functional annotation of pearl oyster *P. fucata* have been performed by our lab [30]. By BLAST analysis of the all annotation sequences (ESTs), a 960 bp fragment of *MSX* gene homologous to *Corbicula fluminea* (AB302955.1, e-value: 1e-66) was obtained. To obtain full-length cDNA of *P. fucata MSX* homolog, 5′and 3′ RACE were performed using SMART RACE cDNA Amplification Kit (Clontech, Japan) following the manufacturer’s instructions. 5′-RACE was performed using gene-specific primers (5′MSX1: 5′*-*TCACCGACTCCGAAACAGG-3′, 5′MSX2: 5′*-*TCTTCTGTCAAGTTCATCCGTG-3′). 3′-RACE was performed using gene-specific primers (3′MSX1: 5′-CGGACGCCATTCACAACGTCAC-3′, 3′MSX2: 5′-CTCAAGTCAAGATTTGGTTTC-3′). The PCR products were purified with Gel Extraction Kits (Omega, USA) following the manufacturer’s instructions and sequenced (Invitrogen, USA). *PfMSX* sequences were analyzed using the BLAST algorithm at NCBI web site. SMART was used to analyze the deduced amino acid sequences of *PfMSX* [31]. The program Bioedit was used to align the MSX sequences and to calculate their sequence similarities [32]. Phylogenetic analysis was performed by MEGA software 4.1 by Neighbor-joining method and 1000 replications of bootstrap [33]. The percentage of similarity to the known MSX sequence was calculated by the Bioedit program. Genomic DNA was isolated from one *P. fucata* adduct muscle using Mollusc DNA Kit (OMEGA, USA) following the instruction manual. Based on the *PfMSX* cDNA sequence, four primers (DMSXF1: CCGTGCGGATATTTGGTGT; DMSXR1: GATCGCAGGTGGTAACATCG; DMSXF2: CGGTTAGTTCAGACGACAGT; DMSXR2: ACAATACATACAAAAGGCGGTG) for *PfMSX* genomic sequence were designed, and four fragments were obtained by PCR and sequenced. The extrons and introns were determined by Splign program [34].

### RNA isolation and quantitative PCR (QPCR) analysis

*P. fucata* samples were isolated using Trizol (Invitrogen, USA). Total RNA (1 µg) was treated with DNase I (Fermentas, China) and subsequently reverse transcribed with TOYOBO RT-PCR kit (TOYOBO, Japan). The QPCR primers for tissue and developmental stages distribution were as follows: PfMSX, 5′-ATGCACCCGGTAGCTCTA-3′ and 5′-TCACCGACTCCGAAACAGG-3′; β-actin, 5′-TGGTATGGGACAGAAGGAC-3′ and 5′-GACAATGCCGTGCTCAAT-3′. QPCR was carried out using a LightCycler 480 Real-Time PCR System (Roche, Basel, Switzerland), with SYBR Green as the fluorescent dye, according to the manufacturer’s protocol (TOYOBO, Japan). QPCR conditions were as follows: denaturation at 94°C for 1 min, followed by 40 cycles at 94°C for 15 s, 55°C for 15 s and 72°C for 60 s. We analyzed the relative gene expression by the typical Ct method (2−^ΔΔCt^ method).

### Cloning of the promoter region of the *Pif* gene

*Pif*-specific primer 1 (5′-TTGTGTCGGTGTCAAATCTG-3′) and nested primer 2 (5′-GCAAGTTCCATCTATTCGAGTTG-3′) were used to clone the promoter of the *Pif* gene by genome walking with the Genome Walker kit (Clontech, USA). The longest fragment from the four genomic libraries (EcoRV, PvuII, StuI and DraI) was gel-purified, and subcloned for sequencing. At least two clones were sequenced, and all were found to be virtually identical in the region directly upstream of the 5′ untranslated region of the *Pif* gene. The *Pif* promoter sequence for the 1358-bp EcoRV fragment was deposited in GenBank [accession no. KJ028207].

### Plasmid constructing

The cDNA encoding the full-length PfMSX was amplified with the sequence specific primers 5′-CGGGGTACCATGCACCCGGTAGCTCTA-3′, containing a KpnI restriction site (underscored), and 5′-CCGCTCGAGATGGTGATACGTCATACCTAC-3′, containing an XhoI restriction site (underscored). After the double digestion with KpnI and XhoI, the cDNA was cloned in-frame into the KpnI/XhoI sites of pcDNA3.1/myc-His (A) vector (Invitrogen,Carlsbad, CA, USA). The construct was verified by sequencing.

A 1358 bp *Pif* promoter fragment (corresponding to bases -1358 to -1) was subcloned into the KpnI and NheI sites of the pGL3basic luciferase reporter vector (Promega, Madison, WI, USA) to generate P1358Luc. The fragments of the oyster *Pif* gene between P1125Luc, P907Luc, P473Luc and P106Luc were amplified by PCR using P1358Luc as a template.

To engineer “ACTAATTGG” or “GTAATTG” deletion mutations, use overlap PCR with the following two sets of primers. The first set was primer A, 5′ -CGGGGTACCGCATGAAGAAAGATCAGGTGATTCTTTA-3′; primer B, 5′-GCTTTTTCATTAAAATTATTTCATA-3′; and primer C, 5′-ATATGACTGGTGGAAAAATATGCAAATA-3′, KpnI site is underscored. “ACTAATTGG” deletion mutation amplified a 126 bp fragment using primer A–B and “GTAATTG” deletion mutation amplified a 727 bp fragment using primer A–C. The second set was primer D, 5′-ATTTTAATGAAAAAGCTACATATATG-3′; primer E, 5′-TATTTGCATATTTTTCCACCAGTCATAT-3′ and primer F, 5′-GCCCGGGCTAGCGACCCTTGTCTTATCT-3′, NheI site is underscored, “ACTAATTGG” deletion mutation amplified a 1027 bp fragment using primer D–F and “GTAATTG” deletion mutation amplified a 419 bp fragment using primer E–F. These PCR products respectively were annealed in equal molar ratios and used as template for PCR with primers A and D. The 1116 bp overlap product of the “ACTAATTGG” deletion mutation and the 1118 bp overlap product of the “GTAATTG” deletion mutation were digested with KpnI and NheI, and ligated to similarly cut pGL3basic separately, creating P1125Δ1Luc or P1125Δ2Luc. The P1125ΔΔLuc created by “ACTAATTGG” and “GTAATTG” deletion mutation was constructed using the same strategy of P1125Δ2Luc basing on the P1125Δ1Luc.

### Subcellular localization

Subcellular localization of PfMSX was performed by EGFP fusion protein expression and immunofluorescence. The 293T cells were seeded onto cover slips (10 mm×10 mm) in a 12-well plate. After the cell adhering for 18 h, the 293T cells were transiently transfected with recombinant pEGFP-PfMSX or empty pEGFP-C1. After transfection for 48 h, cells were washed with PBS (pH 7.4) and fixed with 4% paraformaldehyde for 20 min, and then stained with 6-diamidino-2-pheny-lindole (DAPI) (1 mg/ml) for 10 min. Finally, the cells were rinsed with PBS, mounted with 50% glycerol, and observed using fluorescence microscopy (Leica, Germany). For immunofluorescence localization, the 293T cells were fixed with 4% paraformaldehyde and then the coverslips were blocked using 2% bovine serum albumin (BSA) at room temperature for 30 min. Cells were incubated either with anti-myc antibody (1∶60) or preimmune mouse serum (1∶60) for 1 h, rinsed with PBS three times for 10 min and then incubated with FITC-conjugated goat anti-mouse antibodies (Pierce, USA) for a further hour. Finally, cells were stained with DAPI (1 mg/ml) and observed under fluorescence microscopy.

### PfMSX distribution in *P. fucata*

Adult pearl oysters (shell length 4.5–5.5 cm) were sampled from Daya Bay Station (China Marine Biology Research Station, South China Sea Institute of Oceanology, the Chinese Academy of Sciences) in Shenzhen, China. They were acclimated in indoor cement ponds at ambient seawater temperature for one week before experiment. For *PfMSX* expression analysis in different tissues, three pearl oyster’s ovary, testes, gills, adduct muscle, mantle, heart, digestive gland were collected. For analyzing the developmental expression patterns of *PfMSX*, nine developmental stages including fertilized eggs, 2–4 cell, blastocyst, the trocophore, D-shaped larvae, and umbo larvae, eye-spot larvae, spats and juveniles were collected and stored at −80°C. *β-actin* was used as reference gene which was expressed stably in all tested tissues and developmental stages. Three repetitions of the reaction were performed.

### Cell culture, transfection and luciferase assays

The 293T human kidney cell line (HEK293T), and the C2C12 mouse myoblast cell line were cultured at 37°C in a humidified atmosphere of 5% CO_2_ using DMEM (Gibco, USA) supplemented with 10% FBS (Gibco, USA), 100 IU/ml penicillin and 100 µg/ml streptomycin (Gibco, USA). The cultures were split every 2 to 3 d. Lipofectamine 2000 (Invitrogen, USA) was used for the DNA transfections according to the manufacturer’s protocol.

C2C12 cells grown to 80% confluence in a 48-well plate were transfected with 100ng/well of the reporter construct along with 100 ng/well of pRL-TK plasmid (internal control) in the absence or presence of *PfMSX* expression vectors. At 48 h after transfection, the cultures were harvested and lysed. Luciferase assays were performed using 20 µl of cell extract and 100 µl of luciferin substrate (Promega, USA) using a luminometer.

### Electrophoretic mobility shift and supershift assays (EMSAs)

Nuclear protein extract were isolated using the NE-PER Nuclear and Cytoplasmic Extraction Reagents (Thermo Scientific, USA) from HEK293T cells with or without *PfMSX* gene transfectants. The following complementary oligonucleotides were used: Oligo *Pif* wildtype (wt): 5′-AAAAGCACTAATTGGTACATA-3′, and Oligo *Pif* mutated (mut): 5′-AAAAGCACT**CCA**TGGTACATA-3′. The nucleotide changes were shown in boldface and the MSX element is underlined. Sense and antisense oligonucleotide were 5′ end labeled in Takara Biotechnology (DaLian) CO., LTD. Binding experiments were performed with an electrophoretic mobility shift assay kit (Thermo Scientific, USA) according to the manufacturer’s protocol using 6 µg of nuclear protein and 1 µl of 25 fmol of biotin-labeled wild type oligonucleotides. In the competitive reactions, a 100-fold, 200-fold and 400-fold excess of unlabeled wildtype oligonucleotides were added separately. In the mutant reactions, a 100-fold, 200-fold and 400-fold excess of unlabeled mutant oligonucleotides were added separately. In the supershift experiments, protein extracts were incubated for 15 min with 1–2 µg of the anti-myc antibody (Abmart, China) at room temperature before the addition of the probe. Chemiluminescent detection of biotin DNA on membranes was realized with the Chemi-Doc apparatus (Bio-Rad, USA).

### RNA interference (RNAi) experiments

RNA interference was performed as described in Suzuki et al.[27], with some modifications. The primers used for generating the *PfMSX* dsRNA were GCGTAATACGACTCACTATAGGGAGA TTCATGGATCTCCAAAGACAAT (forward); GCGTAATACGACTCACTATAGGGAGA ATGGTGATACGTCATACCTACG (reverse). The T7 promoter sequence was underlined. A RiboMAX Large Scale RNA Production System (T7) kit (Promega, USA) was used to synthesize and purify the dsRNA. RNase-free DNase I (TAKARA, Japan) was used to digest the template DNA. The dsRNA was diluted to 40 µg/100 µl using PBS. Total RNA from the mantle tissue of each oyster was extracted in day 7 after injection and used to synthesize the first strand cDNA as described above. QPCR was used to quantify the expression levels of *PfMSX and Pif,* where *β-actin* was used as an internal reference. QPCR primers were designed for *PfMSX* and *β-actin* which were the same sequences as that in the distribution experiment above. The *Pif* QPCR primers were from [27]. The shell of each oyster was thoroughly washed with Mili-Q water and air-dried. Shells were cut into pieces and then mounted on the scanner with the inner nacreous surface face-up, sputter-coated with 10-nm-thick gold, and analyzed using scanning electron microscopy (SEM, S-3400N, Hitachi, Japan).

### Statistical analysis

Data were analyzed by one-way analysis of variance (ANOVA) with default parameters or t-Student test to identify differences between groups. Differences were considered statistically significant when p values were lower than 0.05.

## Results

### *PfMSX* encodes a conserved homeodomain protein

**Figure 1:**
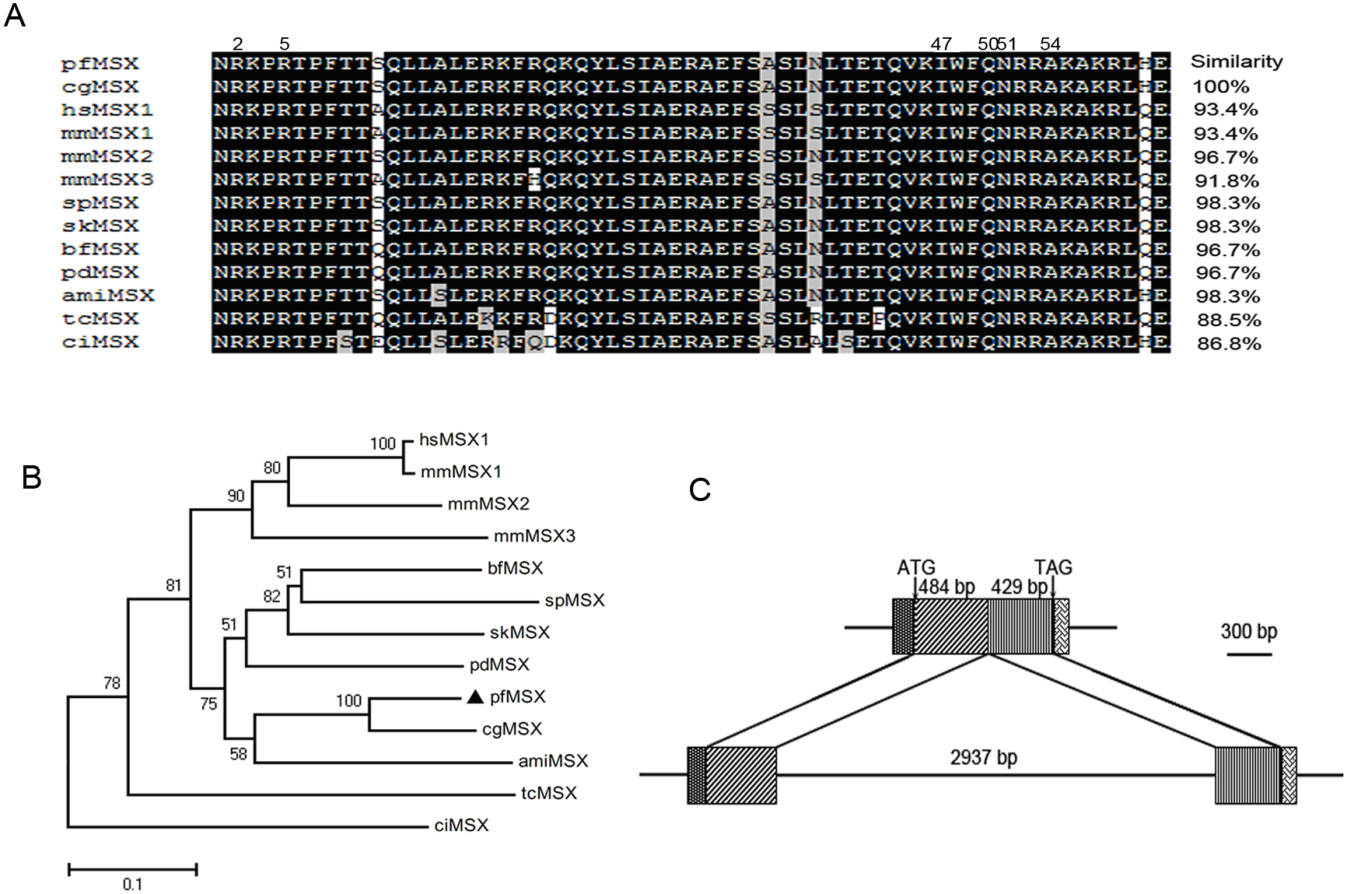
*PfMSX* encodes a homeodomain protein. (**A**) Comparison of PfMSX homeodomain with its homologs. Percentages refer to similarity values between PfMSX and the remaining sequences in the homeodomain region. (**B**) Phylogenetic analysis of the MSX family. The phylogenetic tree was constructed by MEGA 4.1 using the neighbor-joining method with 1000 bootstrap replicates. The number shown at each branch indicates the bootstrap value (%). (**C**) Schematic representation of the structural features of *PfMSX* cDNA and deduced exon/intron organization of *PfMSX*. Filled boxes represent exons and lines between filled boxes represent introns. These MSX amino acid sequences using in the alignment and phylogenetic analysis are from hsMSX1 (*Homo sapiens*, NP_002439.2), mmMSX1 (*Mus musculus*, NP_034965.2), mmMSX2 (*Mus musculus*, NP_038629.2), mmMSX3 (*Mus musculus*, AAI37578.1), bfMSX(*Branchiostoma floridae*, XP_002607933.1), amiMSX(*Acropora millepora*, ABK41269.1), cgMSX(*Crassostrea gigas*, EKC33079.1), spMSX(*Strongylocentrotus purpuratus*, AAB97688.1), skMSX (*Saccoglossus kowalevskii,* ABD97280.1) and ciMSX(*Ciona intestinalis,* CAD56691.1), tcMSX (*Tribolium castaneum,* AAW21975.1).

The full-length of *PfMSX* cDNA, deposited in GenBank under accession no.KJ028206, consisted of a 5′-untranslated region (UTR) of 127 bp, a 3′-UTR of 106 bp, and an open reading frame (ORF) of 912 bp encoding a polypeptide of 304 amino acids with an estimated molecular mass of 33.3 kDa and a theoretical isoelectric point of 9.92. A comparison of the predicted homedomain amino acid sequence with those of a representative set of protostomes and deuterostomes revealed a striking conservation (similarity 86.8%–100%) (Figure 1A). The phylogenetic tree shown (Figure 1B) indicates that the PfMSX have close relationship with that in Vertebrata, Cephalochordata, Hemichordata, Echinodermata, Brachiopoda, and Anthozoa which tallied with Takahashi’s view [15]. Comparison of the cDNA sequence with genomic sequence data [GenBank accession no.KJ028208] revealed that *PfMSX* gene covers 4119 bp and has two exons and one intron. The intron, we call it “AC intron” which was acquired in a common ancestor of a eumetazoa or metazoan [15]. The deduced exon/intron organization was illustrated in Figure 1C.

### PfMSX is localized to the nucleus

**Figure 2:**
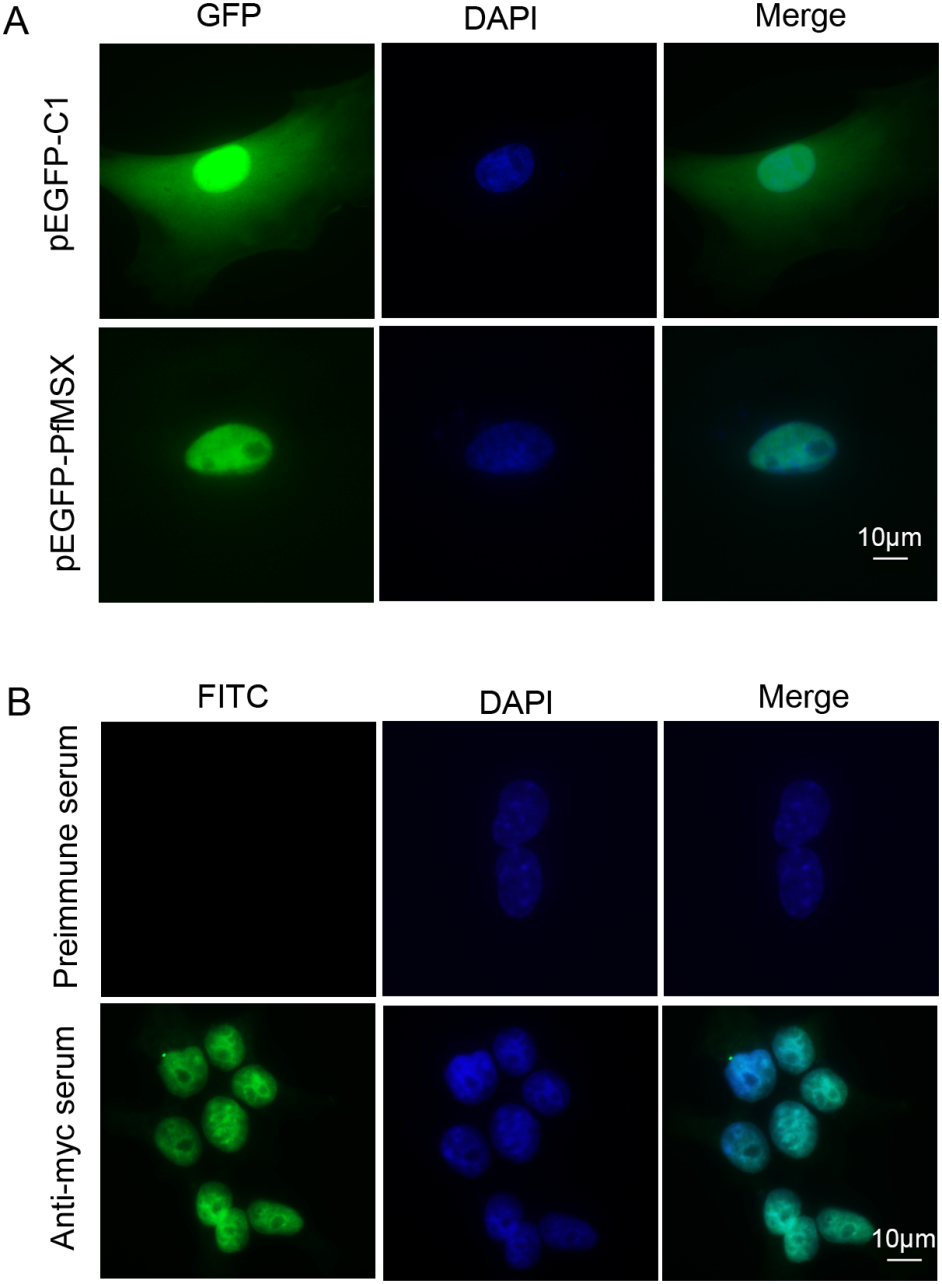
The sub-cellular localization of pfMSX in HEK293T cells. (**A**) Intracellular localization of PfMSX by fluorescence microscopy, 293T cells were transfected with pEGFP-C1 (upper row) or pEGFP-pfMSX (Lower row). The localization of the nucleus was shown by DAPI staining. (**B**) Indirect immunofluorescence staining of PfMSX using mouse anti-myc antibody and FITC-conjugated goat anti-mouse antibodies (lower row). Preimmune mice serum was used as control (upper row), and blue images show the location of the nucleus stained by DAPI.

The subcellular localization of PfMSX was determined by GFP fusion protein expression and immunofluorescence assay in HEK293T cells. As shown in Figure 2, the green fluorescence in PfMSX-GFP fusion protein transfected 293T cells was in the nucleus (Figure 2A, lower row). In pEGFP-C1 transfected cells, the fluorescence signal was observed in both cytoplasm and nucleus (Figure 2A, upper row). Consistently, the immunofluorescence assays showed that the PfMSX mainly accumulated in the nucleus (Figure 2B, lower row). No fluorescence signal was detected in the control cells detected by the preimmune mouse serum (Figure 2B, upper row). The same location results were also found in rat BMSC (bone marrow derived mesenchymal stem cells) cells and C2C12 cells (unpublished data).

### *PfMSX* expression in different tissues and different developmental stages

**Figure 3:**
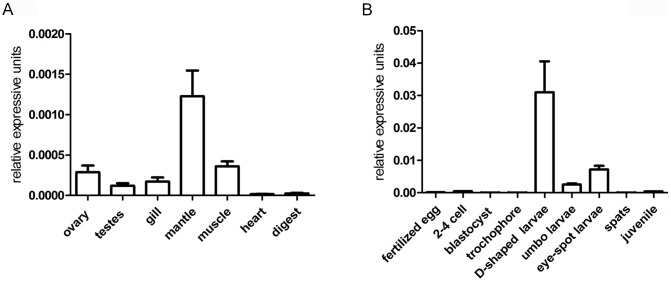
Expression of *PfMSX* mRNA in various tissues (A) and at the developmental stages of *Pinctada fucata* (B). The mRNA levels were quantified by QPCR. The results are expressed as fold-change. Each bar represents the mean ± S.E.M (n = 3).

Gene expression analysis indicated that *PfMSX* mRNA was constitutively expressed in all detected tissues. The expression level of the *PfMSX* in mantle was higher than in other tissues, and the expression in heart and digest was in a lower level (Figure 3A). During the developmental stages, the *PfMSX* expression was in very low level from the fertilized egg to the trocophores, then increased dramatically to a high level at the D-shaped larvae stage, then dropped significantly at the umbo larva stage (Figure 3B).

The mantle tissue is corresponding to the shell formation and the D-shaped larva stage is a period in which mineral materials accumulate largely. These results may suggest *PfMSX* plays an important role in shell formation not only in adult but also during the embryonic stage.

### PfMSX directly binds to the MSX binding site of the *Pif* promoter

**Figure 4:**
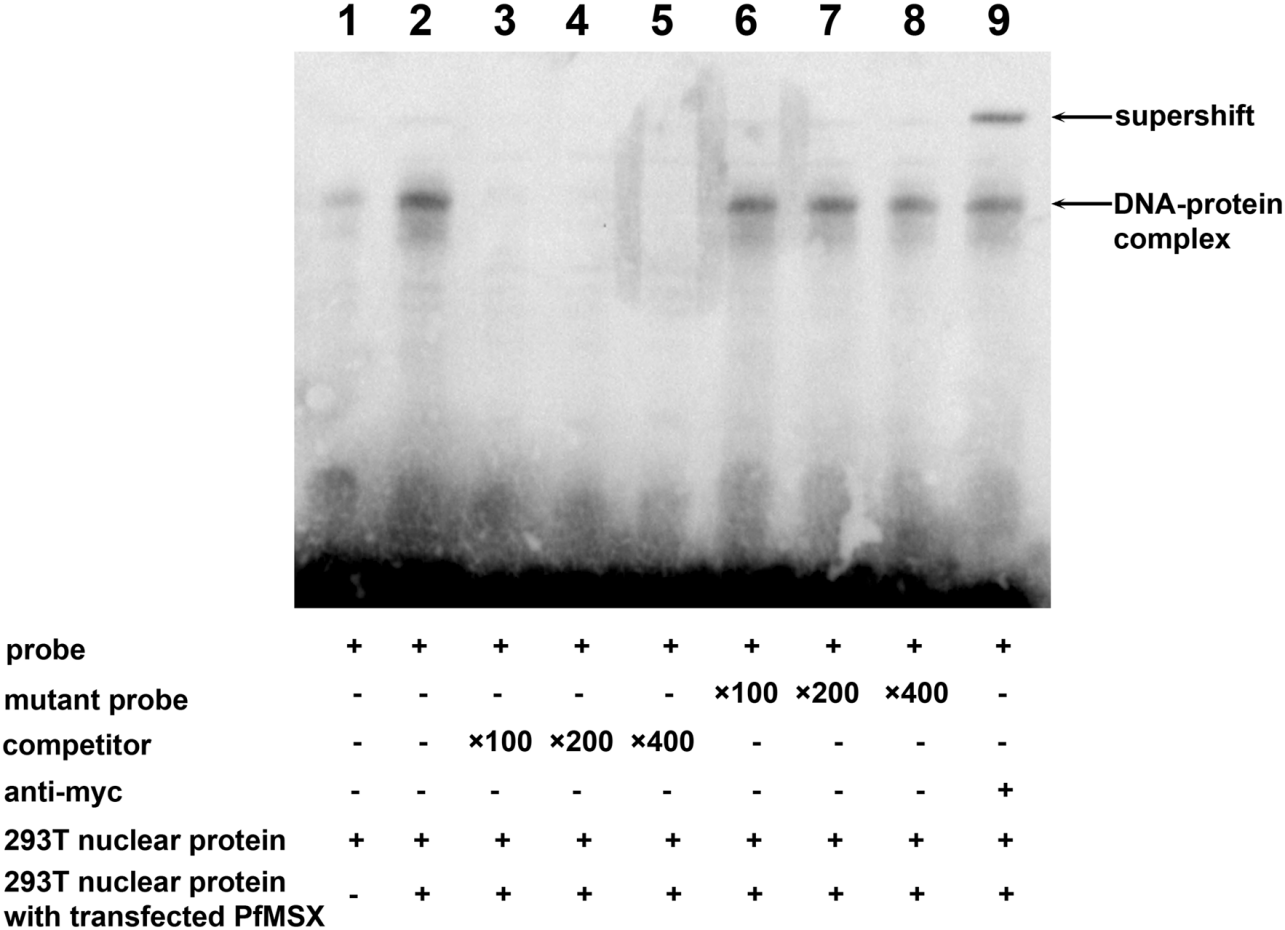
EMSA for binding of PfMSX to the *Pif* promoter region through MBS-1. The labeled probe is incubated with nuclear extract (6 µg) from HEK293 cells without any transfection (lane 1) or transfected with pHis/myc-PfMSX vector (lane 2). The gel retardation complex is indicated by “Protein-DNA complex”. Lane 3–5: competition with 100-fold, 200-fold, 400-fold molar excess of unlabeled probe abolished the complex. Lane 6–8: competition with 100-fold, 200-fold, 400-fold molar excess of unlabeled mutant probe did not disrupt the complex. Lane 9: supershift band was conserved by using the anti-myc antibody. The free probes ran off the gel.

The ability of a protein to bind selectively to a particular DNA site in the genome is the foundation upon which transcriptional regulatory pathways are built [35]. To address the possibility that PfMSX modulates *Pif* expression by binding to MSX binding site of this gene, we performed gel electrophoretic mobility shift assays (EMSAs), using WT and mutated oligonucleotide probes encompassing residues −1000 to −1008 of the pearl oyster *Pif* promoter sequence. Figure 4 *line 1* shows that a faint retardation complex was obtained with HEK293T cell nuclear extract without any transfection, which would relate to the high-expression of MSX1/MSX2 in HEK293T cells (see [36] and protein expression data from MOPED, PaxDb and MAXQB**)**. Figure 4 *line 2* shows that a stron*g* retardation complex with HEK293T nuclear extracts transfected with pHis/Myc-PfMSX construct. The anti-myc antiserum supershifted the PfMSX-wt complex (Figure 4, *lane 9*). When the TAAT core sequence of MSX-binding site in *Pif* promoter was competed with 100-fold, 200-fold, 400-fold excess of the unlabeled DNA fragment, PfMSX binding were abolished (Figure 4, *lane 3–5*). When competed with 100-fold, 200-fold, 400-fold excess of the mutant DNA fragment which the TAAT core was mutated to TCCA (mut), PfMSX binding did not disrupted (Figure 4, *lane 6–8*). These results suggested PfMSX may directly bind to the ACTAATTGG motif, and bind to the TAAT sequence. Since the core sequence of MBS-2 is identical to that of MBS-1, it is reasonable to speculate that PfMSX could bind to MBS-2.

### PfMSX activates Pif specific reporter genes through MBS-1

**Figure 5:**
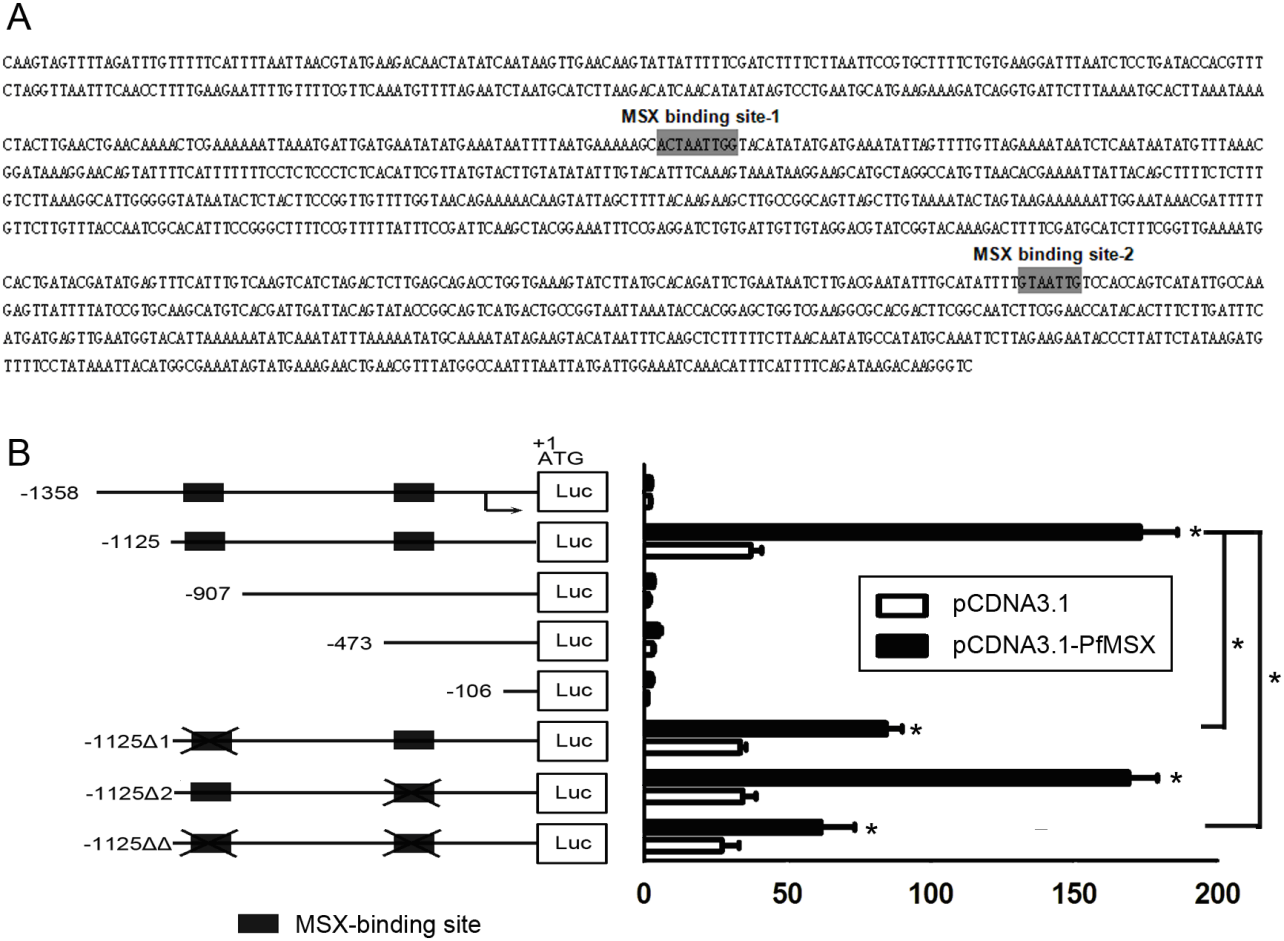
MSX activates *Pif* promoter through MBS-1 in C2C12 cells. (**A**) The fragment of the 5′ flanking sequence of the *Pif* gene, MBS-1 and MBS-2 were grey shaded. (**B**) Left graph: indicated segments from the 5-flanking region of the *Pif* gene with or without MBS-1 and/or MBS-2 linked to PGL3basic encoding luciferase. Right graph: the synthetic Pif-Luc reporter was transfected into C2C12 cells in the absence (vector) or presence of expression vectors for *PfMSX*. 48 h after transfection, whole cell lysates were prepared and analysed for luciferase activity. The bars indicate relative luciferase activity. Normalized luciferase activities are shown as the mean± S.E.M (n = 3). Statistically significant differences were analyzed by means of t-Student test. Asterisk indicates a significant reduction (P<0.05).

To elucidate whether PfMSX activates *Pif* expression, we cloned regulatory elements in the pearl oyster *Pif* 5′-flanking region from −1358 to −1 with a series of 5′ deletion promoter-luciferase constructs (Figure 5B, left graph) and tested their transcriptional activity in C2C12 cells in the present or absent of *PfMSX* vector. P1125Luc is the bsasic promoter of the *Pif* promoter. Deletions of the region from −1358 to −1125 resulted in 20-fold increases in promoter activity, suggesting that these regions function as silencers in controlling *Pif* gene transactivation (Figure 5B, right graph). Recent ChIP-chip and ChIP-seq studies have shown that the number of transcription factor binding sites in vivo is large, but many of them might not function in gene regulation in any particular situation [37]. Since there are two TAAT elements found in the 5′ flanking sequence of the *Pif* gene, to determine which one might be functionally important for PfMSX activating *Pif* expression, *PfMSX* vectors were applicated to activate Pif1125Luc promoter constructs without MBS-1 and/or MBS-2 (Figure 5B, left graph). The basic promoter P1125Luc containing the MBS-1 and-2 had 3.9-fold increases responded to the *PfMSX* vector. Furthermore, deletion of the MBS-1 (P1125LucΔ1), P1125LucΔ1 had 2.8-fold increases; Deletion of the MBS-2 (P1125LucΔ2), the increase was 4.1-fold; Deletion of the both binding site (P1125LucΔΔ), the increases was 2.1-fold. There is significant difference was noted in the ability of P1125Luc construct and P1125LucΔ1 or P1125LucΔΔconstruct to enhance this responsiveness but no significant difference between P1125Luc and P1125LucΔ2 construct (Figure 5B, right graph). These results suggested the PfMSX activated *Pif* specific reporter genes through MBS-1 not MBS-2 and consensus MSX-binding site in *Pif* promoter is a cis-regulatory element.

### Knockdown of PfMSX leads to *Pif* expression reduction and disorder of nacreous layer

**Figure 6:**
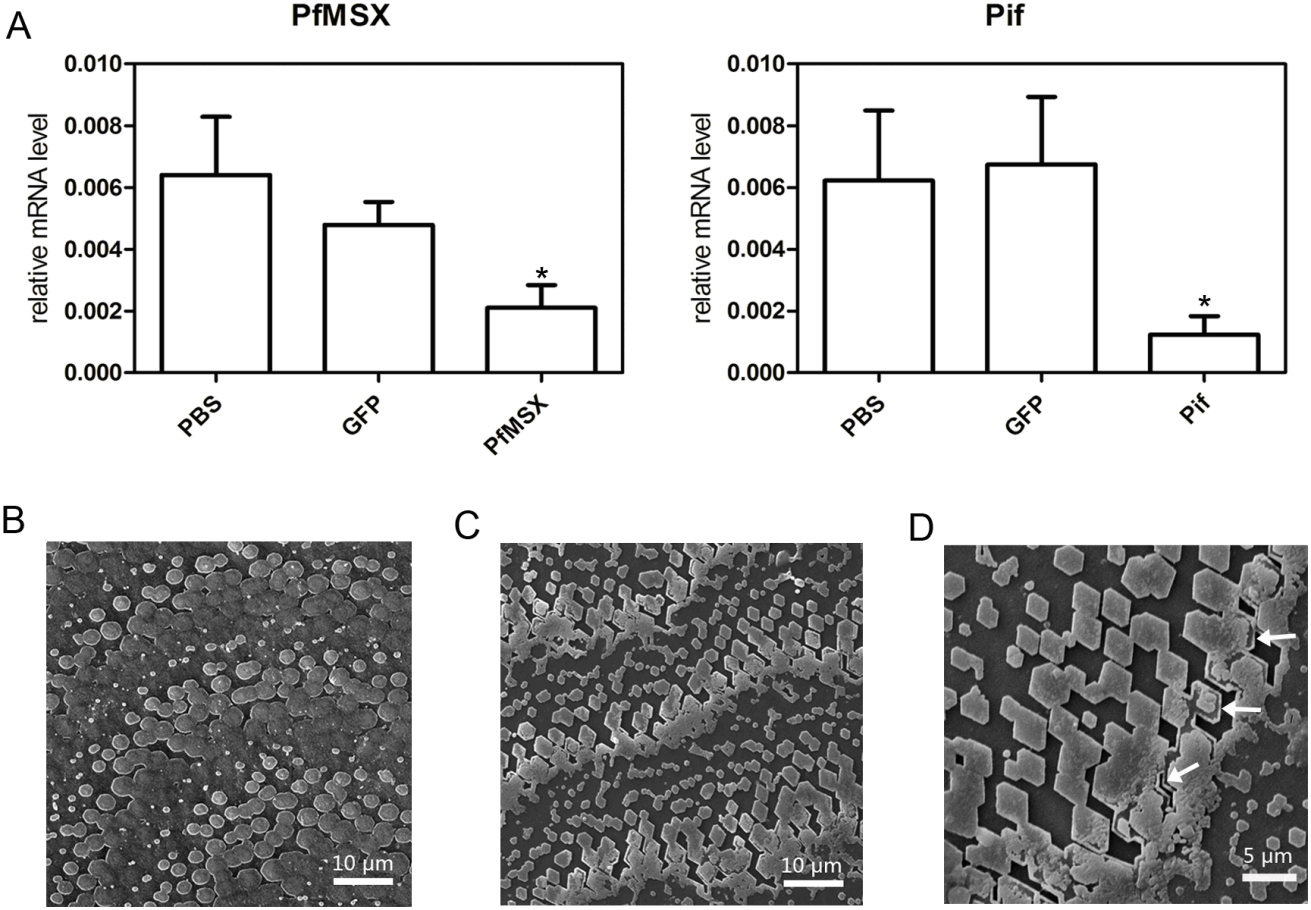
Knockdown of the *PfMSX* gene by means of RNAi. (**A**) The expression levels of *PfMSX* and *Pif* mRNA in the mantle were determined with QPCR 7 days after injection. Five oysters were used in each experiment. Statistically significant differences were analyzed by means of one-way analysis of variance. Asterisk indicates a significant reduction (P<0.05) as compared with PBS-injected oysters. (**B)** and (**C**) SEM images of the surface of the nacreous layer of the oysters injected with PBS and 40 µg of *PfMSX* dsRNA in the magnification of 1000, respectively. (**D**) SEM images of the surface of the nacreous layer of the oysters injected with 40 µg of *PfMSX* dsRNA in the magnification of 1500.

Double-stranded RNA injection in invertebrate has been shown to be an effective tool for interfering with the function of endogenous genes [27], [38], [39]. To clarify the function of PfMSX to shell formation in vivo, double-stranded RNA (dsRNA) designed from the *PfMSX* cDNA sequence was injected into the muscle of *P. fucata*, and the expression levels of *PfMSX* and *Pif* mRNA in the mantle were measured with QPCR 7 days after injection. The *PfMSX* and *Pif* expression level of the group injected with 40 µg of *Pif* dsRNA were suppressed to approximately 60% and 70% of that of the phosphate-buffered saline (PBS) or green fluorescent protein (GFP) dsRNA-injected group respectively (Figure 6A). The surface structure of the nacreous layer in each injection group was observed with SEM. Nacre lamella is composed of polygonal aragonitic tablets which are constituted by nanosized biocrystals of CaCO3. The space between each aragonitic tablet is sealed by intertabular matrix. The normal orderly structure of the nacreous layer was observed in the PBS injected groups (Figure 6B), whereas a disordered growth of the nacreous layer was observed in *PfMSX* dsRNA-injected groups (Figure 6C) and of note, the nacre tablets become scare in the experimental groups and the shape of the nacre tablets changed from quasi-hexagonal to rhomboid (Figure 6C and Figure 6D). On closer inspection,part of aragonite biocrystals was lost in the nacre tablets, underlining the intertabular matrix in the expremental groups (as indicated by the white arrows in Figure 6D). With regard to this phenomenon, we suggested that the knockdown of *PfMSX* caused disorder in CaCO_3_ crystallization, for normal expression of some matrix protein like *Pif* was disturbed. These changes may further affect the iridescent color of nacre which depends on the thickness of the last few layers of nacre and the regularity of the tablets at the surface of the shell [40].

## Discussion

### PfMSX can regulate biomineralization process in the pearl oyster

So far as metazoans, our knowledge of gene regulatory mechanisms involved in biomineralization in invertebrates has been limited compared to that in vertebrates. Up to now, there is no information about transcription factor involved in direct regulation of matrix protein in pearl oyster, and *PfMSX* is the first gene reported here.

Pif is an important component in the nacreous layer and takes part in the initiation of aragonite crystallization as well as subsequent stacking of aragonite tablets in the nacreous layer. *Pif* gene harbors two MSX binding sites which is identical to that in mammals in its promoter. Current MSX appears to have retained an ancient MSX binding site during the long course of evolution. Our data showed PfMSX could bind to the MSX binding site in the promoter of *Pif* gene and then mainly activate its expression through MBS-1 not MBS-2. The MBS-2 seems to be redundancy to *Pif* promoter, actually it implies more. Even in regulatory sequences with highly conserved function, transcription factor binding sites can be gained and lost over time at a high rate, leading to considerable differences in the composition and arrangement of binding sites between even closely related species. It is likely that the redundancy offered by such a system plays a role in avoiding the deleterious effects of uncontrollable mutations [37]. As referred to the *Pif* luciferase promoter being markedly activation by the *PfMSX* vector after deleting both the MSX binding sites (Figure 5B, right graph), it is reasonable to postulate that some other MSX binding sites exiting in the *Pif* promoter which we are not certain exhibit functioning in *Pif* gene regulation. We subsequently investigated the function of PfMSX in vivo by the means of RNAi. The injection of *PfMSX* dsRNA decreased the expression levels of *Pif* mRNA strikingly and at the same time disordered the nacreous layer. The lamellar sheet in nacre surface showed different pattern comparing to that injected with *Pif* dsRNA in the literature, implicating that PfMSX may exert function in other unknown matrix protein in the pearl oyster. Collectively, the MSX of *P. fucata* could activate the expression of *Pif* gene.

The Pacific oyster and the pearl oyster are in close kinship. The genome of the Pacific oyster *Crassostrea gigas* was sequenced recently [41]. Some homeobox-containing genes revealed are involved in the shell formation, which they had special or high expression in mantle tissue (see [41] Supplementary Table S24). Intriguingly, we found a gene among them annotated share similarity with XHOX-7.1 (also referred to as MSX1 in human). This finding is consistent with our data that *PfMSX* has high expression in the mantle tissue.

Taken together, PfMSX takes part in the regulation of biomineralization in pearl oyster.

### Highly conserverd homeodomain and ancestral role in biomineralization

Highly conserved *MSX* genes were identified in the Vertebrata, Cephalochordata, Hemichordata, Echinodermata, Mollusca, Brachiopoda, and Anthozoa [15]. Our data showed PfMSX is similar to chordate MSX sequences and diverged from MSX sequences from Class Ascidiacea, Nematoda, Insecta and Annelida. The wide distribution of the conserved sequences suggested that metazoan ancestors had already acquired a set of conserved domains of the current MSX family genes.

The DNA binding sites of MSX1 proteins have been defined. The canonical TAAT sequence is the core binding site for MSX1 [42]. Residues at positions 2 and 5 of the homeodomain interact with bases in the minor groove, and residues at positions 47, 50, 51, and 54 of the recognition helix are positioned to make contacts in the major groove (the numbers shown in the Figure 1A). Given that the homeodomains of the MSX gene family share high homology and the residues at those positions which interact with bases in the minor groove and major groove are the same to the human MSX1. It is reasonable to speculate that the conserved homeodomain of the MSX gene family could bind to consensus MSX binding site. In our EMSA assays the PfMSX binding to the consensus MSX-binding element and abrogating binding to the mut core, supports the notion.

The highly conserved homeodomian and highly conserved binding site may share some identical features in the course of evolution. For many homeobox genes involved in developmental processes, more and more comparative data are becoming available. From these data, it is evident that in groups of very diverse animals many genes share the same function. There are accumulating examples of MSX orthologs implicating in regulating biomineralization: vertebrate MSX genes are expressed in limb bud, mandibular process, tooth; Zebrafish MSX genes are expressed strongly in fin tissues [43], [44]; the MSX of sea urchin *Strongylocentrotus purpurutus* was abundant in blastula stage and prism stage during which the skeleton formed and the related sea urchin *Heliocidaris tuberculata* injected with a morpholino to *HtMSX* exhibited reduced skeletal growth [45], [46]. These results reinforced by our data that the homeodomain and its conserved binding site exert function in regulating biomineralization.
